# Development of a Communication Strategy to Increase Interprofessional Collaboration in the Outpatient Setting

**DOI:** 10.3390/pharmacy6010004

**Published:** 2018-01-06

**Authors:** Chelsea Phillips Renfro, Stefanie Ferreri, Tiffany Graham Barber, Stephanie Foley

**Affiliations:** 1 Department of Clinical Pharmacy and Translational Science, University of Tennessee Health Science Center College of Pharmacy, Memphis, TN 38163, USA; crenfro@uthsc.edu; 2 UNC Eshelman School of Pharmacy, University of North Carolina at Chapel Hill, Chapel Hill, NC 27599, USA; 3 Hillsborough Pharmacy and Nutrition, Hillsborough, NC 27278, USA; tgrahambarber@gmail.com; 4 UNC Family Medicine at Hillsborough, Hillsborough, NC 27278, USA; Stephanie.Foley@unchealth.unc.edu

**Keywords:** community pharmacy, medication therapy management, electronic health record, patient care team, communication

## Abstract

Managing patient health is a complex task, requiring the support of an interprofessional healthcare team. Collaboration between neighboring community pharmacies and primary care practices can be an alternate solution for team-based patient care. The purpose of this project was to design and implement a communication strategy for patients with diabetes and hypertension between a community pharmacy and physician practice. An interprofessional team for the practice settings was formed to develop a strategy for collaboration. After agreeing on the common goals and target patient population for the disease states, the team devised a way to communicate via electronic health record (EHR). The communication strategy allowed for more frequent follow-up with the patients which has the potential to result in better clinical outcomes. A communication strategy between a community pharmacy and a physician practice office can be achieved using EHR technology. The greatest outcome of this project was the formation of the collaborative team between the practice settings that continues to work together on additional patient-centered initiatives. Further research is warranted to allow for incorporation of patient perspectives in development of communication strategies.

## 1. Introduction

Managing patient health is a complex task, requiring the support of an interprofessional healthcare team. Pharmacists play a pivotal role in optimizing medication regimens with the team. This role is extremely important since approximately 75% of medication related problems deal with inappropriate or ineffective prescribing, lack of care coordination, and inconsistent monitoring in the primary care setting [1]. 

Although a number of successful interprofessional collaborations have been published showing an improvement in patient care and health outcomes [2,3], most are conducted in academic ambulatory practices [4,5,6,7] or in settings where pharmacists and prescribers are in the same building [3,8,9]. However, it may not be feasible for all practices to employ a full-time pharmacist in primary care. In order to fully exploit the benefits of interprofessional collaboration, physicians and pharmacists need to find ways to effectively integrate care, despite the fact that they practice in different settings. Collaboration with neighboring community pharmacies can be an alternate solution for team-based patient care [10]. 

In order to improve care coordination for patients, prescribers and pharmacists practicing in different settings must communicate and collaborate effectively and efficiently to ensure patients receive high quality, patient-centered care [10]. Traditionally, community pharmacies and prescribers communicate via telephone or facsimile, but repeated calls and faxes can create workflow inefficiencies and interruptions. Sharing access to an electronic health record (EHR) is likely to support more efficient communication without creating interruption of the prescribers’ workflow. However, adoption of shared EHR use within community pharmacies is not common. It is unknown whether this communication is more efficient with minimal interruptions [11,12,13].

### Objectives

The purpose of this project was to design and implement a communication strategy utilizing an EHR as the method of communication for shared patients with diabetes and hypertension between a community pharmacy and family medicine practice. The secondary objectives were to assess prescriber acceptance rate and clinical outcomes for those patients.

## 2. Methods

### 2.1. Setting

This descriptive project took place between a single, independent community pharmacy and a single family medicine practice which is associated with an academic medical center’s physician network. Both practices are located in Hillsborough, North Carolina which is an underserved, rural town. 

### 2.2. Practice Description

The community pharmacy offers a large range of clinical services including medication therapy management (MTM) services, immunizations, medication synchronization and adherence packaging. The pharmacy partnered with Community Care of North Carolina and UNC Eshelman School of Pharmacy to serve as a 2-month rotation site for a pharmacy practice resident. The described collaborative project was one of several new projects implemented to improve patient engagement and care.

### 2.3. Communication Strategy Development

To facilitate collaboration between the community pharmacy and the family medicine practice, the community pharmacist and pharmacy resident set up a face-to-face meeting with prescribers and office staff. The purpose of the meeting was to discuss the needs of the practice, goals of collaboration and an effective method for communication. During the face-to-face meeting, the community pharmacy shared clinical services offered and desired goals for patient care. This allowed the practice to determine where the pharmacy’s goals aligned with the practice’s goals. After assessing internal practice performance metrics, it was determined that the practice would benefit most from collaborating with the pharmacy to co-manage patients with diabetes or hypertension. The six shared goals identified by the pharmacy and practice site are in Table 1.

During this meeting it was also established that the most effective method for communication was secure electronic messaging via the practice’s electronic health record (EHR). The family medicine practice belongs to a health-system which uses a shared EHR across all practices. The clinical systems developer for the health-system EHR allows read-only access as well as secure messaging features to non-affiliated, community partners. With help from the practice’s office manager, the pharmacy applied for this access through the health-system and was approved. This access allowed pharmacists at the community pharmacy to view medical records for shared patients; access scheduled appointments, allergies and outpatient and inpatient notes (including admissions, discharges and emergency services); and send and receive secure messages to the practice regarding a shared patient’s care.

The meeting also resulted in the development of an interprofessional project team, including 2 ambulatory care pharmacists, 1 pharmacy resident, 1 community pharmacist, 3 physicians, 1 physician assistant, 1 nurse, and 1 office manager to develop a communication strategy for providing care for patients with diabetes and hypertension. Additional meetings with team occurred at least monthly to develop the strategy. E-mail communication was used to ensure feedback was received from all project team members if they were not available to attend a meeting.

### 2.4. Patient Population Idenfitication

Next, the community pharmacy and the family medicine practice identified patients who would benefit from co-management. The interprofessional project team decided to focus on patients with diabetes and hypertension who were patients at both the community pharmacy and family medicine practice. The practice wanted to focus on this group of patients as they felt this is the area where collaboration between both settings was most needed. Patients with suboptimal blood pressure or hemoglobin A1c as defined in Table 2 may be referred by the prescriber to the pharmacist for co-management.

### 2.5. Communication Strategy Process

Patients were referred to the pharmacy by the prescribers through EHR communication. Upon receiving a referral for co-management, the patient would be evaluated by the pharmacist for an initial comprehensive medication review (CMR). The CMR consisted of a face-to-face visit with the patient and/or patient’s caregiver. During the diabetes focused CMR, the pharmacist would also review the patient’s self-monitoring blood glucose (SMBG) log and establish a SMBG plan if the patient had diabetes. If the patient was being referred for hypertension, the pharmacist would also check blood pressure reading and educate patient on how to check blood pressure at home.

The pharmacist would perform longitudinal follow-up after the initial CMR was completed as deemed necessary. For diabetes focused follow-up, the pharmacist would review the patient’s SMBG log, follow-up on existing/identify new drug therapy problems, and review and promote medication adherence. During hypertension focused follow-up visits, the pharmacist would check the patient’s blood pressure, review home monitoring blood pressure readings if patient was checking at home, follow-up on existing/identify new drug therapy problems, and review and promote medication adherence.

At the conclusion of each visit, the pharmacist would send the prescriber a visit summary with a list of medication variances and noted concerns about adherence to their medication regimen through EHR communication no more than 2 days after the visit. This communication included recommendations for change or recommendations regarding any new or existing drug therapy problems, when applicable. All recommendations made were based on either the American Society of Hypertension and the International Society of Hypertension guidelines [17] or American Diabetes Association Standards of Care [14]. Pharmacists obtained approval from the prescriber prior to making any medication regimen changes.

The prescriber at the family medicine practice responded to the pharmacists’ recommendations by either sending a new prescription to the pharmacy or by responding to the EHR secure message. The care provided by the community pharmacist was designed to complement, not replace, the usual and standard care provided by the patient’s prescriber, therefore pharmacists continued to follow-up with patients in between prescriber visits and communicate findings with the family medicine practice.

An emergency plan was also developed for patients with hypertension and diabetes. If a patient presented with hypertensive urgency, BP > 180/110 mmHg, the community pharmacist would call the family medicine practice to schedule a same day appointment for the patient to be seen for evaluation. If a patient presented with hypertensive emergency, BP > 180/110 mmHg with symptoms of end organ damage, the community pharmacist would call 911 for the patient to receive emergency medical attention. The pharmacist would also contact the family medicine practice regarding the patient’s condition.

For patients with diabetes, patients who presented to the pharmacy would have his/her blood glucose tested. If the blood glucose was <70 mg/dL, the community pharmacist would provide the patient with 4 glucose tablets and have the patient recheck blood glucose in 15 min. If blood glucose was not >80 mg/dL after 3 times or it the patient became unresponsive at any time, the community pharmacist would call 911 for patient to receive emergency medical attention. The pharmacist would also contact the family medicine practice regarding the patient’s condition. If the patient presented with symptoms of diabetic ketoacidosis, the community pharmacist would call 911 for patient to receive emergency medical attention. The pharmacist would also contact the family medicine practice regarding the patient’s condition.

### 2.6. Community Pharmacy and Family Medicine Practice Training

A written strategy was developed by the pharmacy resident with input from the whole interprofessional project team. The strategy included shared goals, target patient population, description of service, emergency plan, template for prescriber referral, template for diabetes and hypertension focused CMR, patient self-monitoring logs for blood glucose and blood pressure, and template for visit summary for prescriber.

All staff at family medicine practice were provided training on the communication strategy and documentation process. The pharmacy resident also provided training to the community pharmacist on the strategy, how to navigate the EHR, and documentation process. All staff at the community pharmacy was made aware of the communication strategy, however specific training was not provided.

### 2.7. Project Evaluation

The project evaluated the average number of recommendations made to the prescribers per patient and the acceptance rate for each of those measures. Recommendations to prescribers were tracked by receipt of a new prescription to the pharmacy or the prescriber responding the secure message in the EHR system. Recommendations were de-identified, collected in Microsoft Excel and analyzed using descriptive statistics. 

Secondary outcomes evaluated included change in hemoglobin A1c levels and blood pressure reading. Hemoglobin A1c levels and blood pressure readings were gathered at baseline and three months after initial CMR with the pharmacist. Hemoglobin A1c levels and blood pressure readings were de-identified, collected in Microsoft Excel and analyzed using descriptive statistics.

## 3. Results

A total of three patients were referred to the community pharmacy through the EHR communication strategy during the first month of the project. Two patients had a diagnosis of type 2 diabetes mellitus and one patient had a diagnosis of hypertension. Baseline hemoglobin A1c readings were 10.1% and 9.4%. At each patient’s three-month follow-up with the primary care provider, both patients’ hemoglobin A1c values decreased to 9.5% and 8.6% respectively. For the patient with hypertension, the baseline blood pressure reading was 208/94 mmHg. After three months, the patient’s blood pressure decreased to 147/72 mmHg. Pharmacists provided an average of four recommendations to prescribers for each patient. One hundred percent of recommendations made to prescribers were accepted. 

The most successful result of this program implementation was the successful development of a communication strategy between a community pharmacy and family medicine practice. Table 3 describes the collaboration steps and approximate time frame to complete each step. Based on positive feedback from prescribers and community pharmacists, a meeting has been scheduled to incorporate more disease states.

## 4. Discussion

Although the collaboration described is in the early stages, it successfully showcases the design and implementation of an interprofessional collaboration between a community pharmacy and a family medicine practice located in separate buildings using EHR technology for communication among shared patients. Due to a primary care provider shortage in rural areas, community pharmacists could play a larger role in improving patient care compared to urban areas [16]. This experience demonstrated that community pharmacists have the potential to be integral members of the interprofessional healthcare team through utilizing bi-directional communication via EHR and longitudinal patient follow-up.

The acceptance rate for medication therapy management (MTM) ranges from 30% to 60% [18,19,20,21]. Prescriber resistance has been reported as one of the biggest barriers to providing MTM services [22,23]. During this project, community pharmacists and the family medicine practice took time to have a sit down, in person meeting to get to know each other and know what services the pharmacy and practice had to offer. This allowed the practice to know what the goals of the pharmacy were and realize where the pharmacy and practice goals aligned. Through this process, the pharmacy was able to obtain provider buy-in to avoid prescriber resistance when making recommendations. 

One challenge to coordinate patient care between community pharmacy and prescribers in the outpatient setting is communication [10]. This challenge was overcome in the implementation of this project by utilizing EHR technology. Community pharmacists and prescribers both believed that this communication mechanism allowed them to share information that the other provider might not have access to. For example, the community pharmacist was able to share prescription fill history for a patient that the prescriber was having difficulty managing. The following quote provides the perspective of one of the physicians from the pharmacy medicine practice on improving the ability to share information: “We had a patient who was a poorly controlled diabetic. I kept augmenting insulin, and it didn’t really matter what I did because nothing was working. It turns out he wasn’t filling his diabetes medication except for when it was time for an office visit. He would fill them then so he could bring in his pill bottles and show me that he had his medication. The community pharmacy had access to his billing record, of course, so when I talked to them, we demystified a lot of things.”

Establishing read-only EHR access for the community pharmacy did not have any cost associated with implementation. The use of this technology to improve interprofessional communication and collaboration could be financially viable through mechanisms such as chronic care management services for Medicare beneficiaries who have two or more chronic health conditions [24]. Collaborative partnerships similar to the one described in this manuscript could lead to improved patient outcomes and potentially higher reimbursement for the practice and the pharmacy. However, this financial impact is yet to be determined. 

Prescribers at the family medicine practice were in full support of the implementation of the communication strategy. However, they weren’t sure how to properly document instances in which the patient chose follow-up at the community pharmacy rather than being required to do so. This concern was addressed by providing education to the providers on how to discuss with the patient different options available for pharmacies in the area and the services available for each and document the conversation in the EHR. 

Due to the early success of this program, the community pharmacy has been able to obtain access to another health-system’s EHR and a meeting with a different outpatient practice has been scheduled. This success is being shared with other pharmacies in North Carolina which has led to these pharmacies actively seeking read-only EHR access so they can implement similar communication strategies. A potential barrier to obtaining access to different health-systems’ EHRs would be that community pharmacists would have to log into each separate EHR. This could cause disruption in the community pharmacy’s workflow. Integration of community pharmacies into health information exchanges would allow for patient information to be received and submitted through an electronic data exchange with current workflow processes [25]. This would alleviate the barrier of obtaining access to multiple different EHRs.

## 5. Limitations

The main limitation in the development of the communication strategy is the lack of patient involvement. Creating an interprofessional project team allowed for an understanding of provider perceptions regarding effective and efficient strategies to provide collaborative care. It did not allow for an understanding of patient perspectives. Further research is warranted to allow for incorporation of patient perspectives in development of communication strategies.

## 6. Conclusions

This project describes the design and implementation of a community pharmacy-family medicine practice collaboration for patients with either diabetes or hypertension. The greatest outcome of this project was the formation of a collaborative team between community pharmacy and family medicine practice that continues to work together on additional patient-centered initiatives. 

## Figures and Tables

**Table 1:** Shared Goals Between Community Pharmacy and Family Medicine Practice.

Shared Goals
1. Identify patients with a diagnosis of hypertension or diabetes that are not at goal.
2. Improve patient outcomes by optimizing a patient’s hypertension or diabetes regimen by monitoring outcomes, adherence, and side effects to prescribed medications/therapies.
3. Enhance patient/caregiver understanding of the prescribed medication regimen and self-management.
4. Formulate recommendations to optimize therapy and decrease health care costs.
5. Assess and triage potential and significant adverse drug reactions and report them to providers.
6. Increase collaboration between the community pharmacy and family medicine practice.

**Table 2:** Patient Identification Criteria for Co-Management.

Hypertension Patient Identification Criteria	Diabetes Patient Identification Criteria
1. Blood pressure >140/90 mmHg despite treatment with anti-hypertensive medications for more than 30 days [14]	1. Hemoglobin A1c > 7% despite treatment with anti-hyperglycemic medications for more than 30 days [15]
2. Patient with a newly prescribed anti-hypertensive medication(s)	2. Fasting blood glucose (FBG) > 130 mg/dL or post prandial blood glucose (PPBG) > 180 mg/dL, indicating uncontrolled diabetes [15]
3. Blood pressure reading of >160/100 mmHg, indicating a classification of “Stage 2 hypertension” [14]	3. Suboptimal adherence [16] to prescribed anti-hyperglycemia medications
4. Suboptimal adherence [16] to prescribed anti-hypertensive medication(s)	4. Patient with a newly prescribed an anti-hyperglycemia medication(s)
5. Prescribed anti-hypertensive medication(s) that may require close monitoring	5. Prescribed anti-hyperglycemia medication(s) that may require close monitoring

**Table 3:** Collaboration steps and approximate time frame.

Collaboration Step	Time Frame
1. Conducted face-to-face meeting with community pharmacy and family medicine practice to identify collaboration opportunities	1 h
2. Identification of shared goals	1 week
3. Identification of shared patients	2 weeks
4. Identification of preferred communication method	2 weeks
5. Identification of an interprofessional team to develop a communication strategy for co-management of shared patients	1 week
6. Development of written communication strategy	1 month
7. Received feedback from community pharmacy and family medicine practice on written communication strategy	2 weeks
8. Revised and finalized written communication strategy	2 weeks
9. Training for community pharmacy and family medicine practice staff on communication strategy	1 month
10. Development of quality improvement processes to asses collaboration	Continuous
